# Cell layer-specific distribution of transiently expressed barley ESCRT-III component HvVPS60 in developing barley endosperm

**DOI:** 10.1007/s00709-015-0798-1

**Published:** 2015-03-22

**Authors:** Julia Hilscher, Eszter Kapusi, Eva Stoger, Verena Ibl

**Affiliations:** Department of Applied Genetics and Cell Biology, Division of Molecular Cell Biology and Glycobiotechnology, University of Natural Resources and Life Sciences, Muthgasse 18, 1190 Vienna, Austria

**Keywords:** ESCRT-III, Barley, Endosperm, VPS60, Cell layer-specific, Seed

## Abstract

The significance of the endosomal sorting complexes required for transport (ESCRT)-III in cereal endosperm has been shown by the identification of the recessive mutant supernumerary aleurone layer1 (SAL1) in maize. ESCRT-III is indispensable in the final membrane fission step during biogenesis of multivesicular bodies (MVBs), responsible for protein sorting to vacuoles and to the cell surface. Here, we annotated barley ESCRT-III members in the (model) crop *Hordeum vulgare* and show that all identified members are expressed in developing barley endosperm. We used fluorescently tagged core ESCRT-III members HvSNF7a/CHMP4 and HvVPS24/CHMP3 and the associated ESCRT-III component HvVPS60a/CHMP5 for transient localization studies in barley endosperm. In vivo confocal microscopic analyses show that the localization of recombinantly expressed HvSNF7a, HvVPS24 and HvVPS60a differs within barley endosperm. Whereas HvSNF7a induces large agglomerations, HvVPS24 shows mainly cytosolic localization in aleurone and subaleurone. In contrast, HvVPS60a localizes strongly at the plasma membrane in aleurone. In subaleurone, HvVPS60a was found to a lesser extent at the plasma membrane and at vacuolar membranes. These results indicate that the steady-state association of ESCRT-III may be influenced by cell layer-specific protein deposition or trafficking and remodelling of the endomembrane system in endosperm. We show that sorting of an artificially mono-ubiquitinated *Arabidopsis* plasma membrane protein is inhibited by HvVPS60a in aleurone. The involvement of HvVPS60a in different cell layer-specific trafficking pathways, reflected by localization of HvVPS60a at the plasma membrane in aleurone and at the PSV membrane in subaleurone, is discussed.

**Electronic supplementary material:**

The online version of this article (doi:10.1007/s00709-015-0798-1) contains supplementary material, which is available to authorized users.

## Introduction

In cereals, the endosperm supports the germinating seedling by providing stored nitrogen, carbon and minerals. The fertilized triploid central cell grows into a storage organ where endosperm transfer cells, embryo surrounding tissue cells, aleurone and starchy endosperm (SE) cells carry out specialized functions at different stages of seed development. SE cells accumulate starch and storage proteins (SP) which are mobilized during seed germination via the release of hydrolytic enzymes by the aleurone layer (Olsen 2004). Both SE cells and aleurone cells contain protein storage vacuoles (PSVs). Together with albumins and globulins, prolamins form the major protein component of most cereal grains and are deposited mainly in subaleurone cells (Galili 2004; Shewry et al. 1995). Seed storage proteins (SSPs) reach their final destination by two main routes: soluble albumins and globulins travel through ER and Golgi to PSVs, whereas most prolamins accumulate in ER-derived protein bodies (PBs). Post-Golgi transport of storage proteins towards the vacuole involves dense vesicles and multivesicular bodies (MVBs) (Ibl and Stoger 2012). SSP transport routes depend on the cereal species, endosperm tissue layer and developmental timepoint (Ibl and Stoger 2012; Zheng and Wang 2014), and some PBs are ultimately deposited in the PSV after bypassing the Golgi. The massive and complex SSP transport in the endosperm is accompanied by extensive reorganization of the endomembrane system during development (Hoh et al. 1995; Ibl and Stoger 2012; Wang et al. 2010).

In maize, *Supernumerary aleurone layer1* (*Sal1*) was found to restrict aleurone cell identity to the outer cell layer of endosperm (Shen et al. 2003). *Sal1* encodes the maize homolog of endosomal sorting complexes required for transport (ESCRT)-III component Vacuolar Protein Sorting46/Charged Multivesicular Body Protein1 (VPS46/CHMP1). It is thought that SAL1 maintains the proper plasma membrane concentration of DEFECTIVE KERNEL1 (DEK1) and CRINKLY4 (CR4), both involved in aleurone cell fate specification, by internalization and degradation of SAL1-positive endosomes (Tian et al. 2007). A *vps22* (ESCRT-II) mutant in rice endosperm showed seedling lethality and severe reduction in shoot and root growth correlating with the formation of a chalky endosperm. Thus, OsVps22 is supposed to be required for seedling viability and grain filling in rice (Zhang et al. 2013). This is in agreement with electronic northern analyses revealing that the expression levels of most of the ESCRT genes were highest in seed-specific tissues (seed coat and endosperm) of *Arabidopsis* (Richardson and Mullen 2011). ESCRT originally refers to a protein-protein interaction network in yeast and metazoan cells that coordinates sorting of ubiquitinated membrane proteins into intraluminal vesicles (ILVs) of the MVB (Babst et al. 2002a; Babst et al. 2002b; Katzmann et al. 2001). MVBs then either fuse with lysosomes/vacuoles leading to degradation of lipids and protein content of ILVs in the vacuolar lumen or they fuse with the plasma membrane, discharging ILVs as exosomes. ESCRT-0, I and II function early in the pathway and are sequentially recruited to endosomes as preformed, stable heteromeric complexes, thereby collecting and concentrating ubiquitinated membrane proteins (Hurley and Emr 2006; Teis et al. 2010; Williams and Urbe 2007). ESCRT-III is necessary for membrane remodelling that drives the biogenesis of MVBs and is further involved in budding of enveloped viruses, abscission of the plasma membrane during cytokinesis, plasma membrane repair (all requiring a topologically similar membrane fission event for budding away from the cytoplasm), surveillance of nuclear pore complex assembly and autophagy (Boura et al. 2012; Buchkovich et al. 2013; Caballe and Martin-Serrano 2011; Carlton and Martin-Serrano 2009; Henne et al. 2013; Hurley and Hanson 2010; Jimenez et al. 2014; Peel et al. 2011; Roxrud et al. 2010; Rusten and Stenmark 2009; Webster et al. 2014). Notably, ESCRT-III is also present in Archaea which lack an endomembrane system, supporting the functional importance of ESCRT-III together with its associated proteins for central aspects of budding processes (Ettema and Bernander 2009; Lindas et al. 2008; Samson and Bell 2009). Whereas ESCRT-III subunits are inactive monomers in the cytoplasm, at membranes, they assemble in a highly ordered manner to generate the transient 450-kDa ESCRT-III complex (Babst et al. 2002a; Teis et al. 2008). The ESCRT-III core complex consists of four subunits which are arranged in two distinct subcomplexes, the Vps20-Snf7 and the Vps2-Vps24 subcomplexes (Babst et al. 2002a). VPS60 and VPS46 are associated proteins and implicated in modulating VPS4 ATPase activity that regulates ESCRT-III disassembly (Hanson and Cashikar 2012). In plants, a cross-species comparison analysis showed for the first time that most ESCRT proteins are present in *Arabidopsis thaliana* and rice (Winter and Hauser 2006). Since then, intensive *in planta* research has been conducted to explore the identity, and the structural and functional characteristics of ESCRT proteins in various tissues of rice, maize and *Arabidopsis* (Cai et al. 2014; Ibl et al. 2012; Katsiarimpa et al. 2011; Katsiarimpa et al. 2013; Katsiarimpa et al. 2014; Korbei et al. 2013; Moulinier-Anzola et al. 2014; Reyes et al. 2014; Richardson et al. 2011; Richardson and Mullen 2011; Scheuring et al. 2012; Shahriari et al. 2011; Spallek et al. 2013; Spitzer et al. 2009; Spitzer et al. 2006; Zhang et al. 2013).

The fact that ESCRT-III acts in ILV sorting on MVBs—which represent central hubs in protein sorting to the vacuole and the cell surface—together with the mutant phenotype of sal1 in seed development prompted us to speculate that ESCRT-III may have a pivotal role for cellular homeostasis of protein trafficking and endomembrane dynamics in cereal endosperm. We therefore set out to transfer knowledge from other plant species to the emerging model crop *Hordeum vulgare* (Saisho and Takeda 2011) with a focus on endosperm, a specialized storage organ and widely used platform for recombinant protein production (Boothe et al. 2010; Magnusdottir et al. 2013; Rademacher et al. 2009; Stoger et al. 2005). We have annotated ESCRT-III members in barley, wheat, rice and maize, and we show that all identified members are expressed during barley endosperm development. Using fluorescently tagged core ESCRT-III components HvSNF7a/CHMP4 and HvVPS24/CHMP3 and the associated ESCRT-III component HvVPS60a/CHMP5 for transient overexpression and localization studies in barley endosperm, we showed by in vivo confocal microscopy localization in the cytosol, at small agglomerations or at the plasma membrane, respectively. Notably, the subcellular localization of HvVPS60a differs between aleurone and subaleurone and the here shown effect of HvVPS60a on protein sorting of an MVB cargo reporter in aleurone may reflect the involvement of HvVPS60a in diverse trafficking pathways depending on the cell layer.

## Material and methods

### Plant material and growth conditions

Barley wild-type variety Golden Promise (GP) and its transgenic derivative TIP3-GFP was cultivated as in (Ibl et al. 2014). OsTIP3::TIP3-GFP construct was a gift of Yasushi Kawagoe and is described in (Ibl et al. 2014; Onda et al. 2009).

### Database searches for ESCRT-III members in cereals

Using known protein sequences of ESCRT-III members of *A. thaliana* (Winter and Hauser 2006), TBLASTN searches for full-length complementary DNA (cDNA) sequences and/or EST contigs of *H. vulgare*, *Triticum aestivum*, *Zea mays* and *Oryza sativa* Japonica were performed using GenBank at NCBI and following databases: *H. vulgare*, IPK Barley BLAST Server (http://webblast.ipk-gatersleben.de/barley/); *T. aestivum*, KOMUGI/Wheat genetic Resources database, Committee for the National BioResource Project Japan (http://www.shigen.nig.ac.jp/wheat/komugi/blast/blast.jsp); *Z. mays*, POPcorn portal (Cannon et al. 2011) and *O. sativa*, DFCI Rice Gene Index (http://compbio.dfci.harvard.edu/cgi-bin/tgi/gimain.pl?gudb=rice). Predicted amino acid sequences were aligned by MUSCLE, and a neighbour joining (NJ) tree (based on aligned sequences with excluding gaps) was constructed to check for consistency of partitioning of proteins to the different ESCRT-III subunits. Both alignment and tree construction were done by programmes implemented in MEGA6 (Tamura et al. 2013). cDNA/EST contig entries coding for proteins with N-terminal or C-terminal deletions or extensions were excluded.

Genomic locations/identifiers for ESCRT-III members which are not annotated in GenBank entries were identified for *Z. mays* (Maize B73 RefGen_v2), *O. sativa* and *H. vulgare* by TBLASTN queries of cDNA to genomic sequences at Ensembl Plants platform or, where unsuccessful for barley and maize, at IPK Barley BLAST Server and POPcorn portal, respectively.

### RNA isolation and RT-PCR

Palea and lemma were removed from 10 to 12 days after pollination (dap) wild-type barley caryopses (Golden Promise) and the embryo cut off. The endosperm was squeezed out of the surrounding pericarp, resulting in sampled endosperm with patches of attached aleurone layer. One hundred milligrams tissue (~12–15 sampled caryopses) was ground in liquid N_2_, and RNA was isolated following the modified Li and Trick protocol (Li and Trick 2005) of Mornkham et al. (2013). Two micrograms of RNA was reverse transcribed using First Strand cDNA Synthesis Kit (Thermo Scientific). Gene-specific primers spanned introns derived from alignment of cDNA/EST information against genomic DNA (Table 1, Supplemental Table 1). PCR fragments were separated on 1.5 % agarose gels stained with EtBr. Primers used and expected lengths of fragments are listed in Table S2.

**Table 1:** Inventory of evidently transcribed ESCRT-III members in *Hordeum vulgare*, *Triticum aestivum*, *Oryza sativa* ssp. japonica and *Zea mays*

ᅟ	Name	Accession number			
		Full-length cDNA/EST contig	Derived protein	aa	Genomic identifier ^g^
VPS2/CHMP2					
*H. vulgare* ^h^	VPS2.1	AK250448	–	224	MLOC_67016
	VPS2.2	AK375733	BAK06928	229	MLOC_10666 ^f^
	VPS2.3	AK368215	BAJ99418	212	MLOC_16800 ^f^
*T. aestivum*	VPS2.1	AK330978	–	224	nd
	VPS2.2	Contig14460 ^a^	–	229	nd
	VPS2.3	Contig17212 ^a^	–	212	nd
*O. sativa*	VPS2.1a	NM_001075069	NP_001068537	224	Os11g0703400
	VPS2.1b	NM_001065783	NP_001059248	225	Os07g0236800
	VPS2.2	NM_001057274	NP_001050739	229	Os03g0639800
	VPS2.3	NM_001071383	NP_001064848	212	Os10g0476400
*Z. mays*	CHMP2.1a	EU969317	ACG41435	224	Scaffold 340 ^ef^
	CHMP2.1b	NM_001139057	NP_001132529	223	GRMZM2G431900
	CHMP2.2	NM_001157066	NP_001150538	229	GRMZM2G046676
	CHMP2.3	NM_001155163	NP_001148635	212	GRMZM2G004996
*A. thaliana*	VPS2.1	NM_126650	NP_565336	225	At2g06530
	VPS2.2	NM_123823	NP_199269	222	At5g44560
	VPS2.3	NM_100276	NP_563696	210	At1g03950
VPS 24/CHMP3					
*H. vulgare* ^h^	VPS24	AK366876	BAJ98079	230	MLOC_73329
*T. aestivum*	VPS24	AK333579	–	230	nd
*O. sativa*	VPS24a	AK242193	–	231	Os03g01810
	VPS24b	TC516772 ^b^	–	229	Os07g29630
*Z. mays*	CHMP3a	EU953837	ACG25955	228	GRMZM2G042552
	CHMP3b	EU956407	ACG28525	229	GRMZM2G165195
*A. thaliana*	VPS24.1	NM_122201	NP_197686	229	At5g22950
	VPS24.2	NM_114369	NP_190086	200	At3g45000
VPS20/CHMP6					
*H. vulgare* ^h^	VPS20	AK357563	BAJ88777	229	MLOC_30872^i^
*T. aestivum*	VPS20	Contig13879 ^a^	–	229	–
*O. sativa*	VPS20	NM_001053857	NP_001047322	228	Os02g0596500
*Z. mays*	CHMP6	NM_001143085	NP_001136557	229	GRMZM2G122983
*A. thaliana*	VPS20.1	NM_125783	NP_568980	219	At5g63880
	VPS20.2	NM_120962	NP_196488	216	At5g09260
SNF7/CHMP4					
*H. vulgare* ^h^	SNF7a	AK376941	BAK08135	220	MLOC_42957 ^f^
	SNF7b	AK354835	BAJ86054	222	MLOC_16305 ^f^
	SNF7c	AK366149	BAJ97352	218	MLOC_17778 ^f^
*T. aestivum*	SNF7a	AK333051	–	220	nd
	SNF7b	AK331201	–	222	nd
	SNF7c	Contig9162 ^a^	–	217	nd
*O. sativa*	SNF7a	NM_001069258	NP_001062723	220	Os09g0267600
	SNF7b	NM_001064575	NP_001058040	220	Os06g0608500
	SNF7c	NM_001072148	NP_001065616	216	Os11g0123500
*Z. mays*	CHMP4a	NM_001254842	NP_001241771	222	GRMZM2G107757
	CHMP4b	EU975735	ACG47853	226	GRMZM2G044805
	CHMP4c	TC482666 ^d^	–	223	GRMZM2G103217
*A. thaliana*	SNF7.1/VPS32.2	NM_119060	NP_194645	219	At4g29160
	SNF7.2/VPS32.1	NM_127541	NP_179573	213	At2g19830
VPS46/CHMP1					
*H. vulgare* ^h^	SAL1 (Tian et al., 2007)	AK252514	ABW81400	204	MLOC_57384
*T. aestivum*	VPS46	AK335766	–	204	nd
*O. sativa*	VPS46	NM_001064712	NP_001058177	205	Os06g0643300
*Z. mays*	SAL1 (Shen et al., 2003)	NM_001111748	NP_001105218	204	GRMZM2G117935
*A. thaliana*	CHMP1A, VPS46.2	NM_105961	NP_565053	203	At1g73030
	CHMP1B, VPS46.1	NM_101635	NP_173215	203	At1g17730
VPS60/CHMP5					
*H. vulgare* ^h^	VPS60a	AK372130	BAK03328	232	MLOC5296
	VPS60b	AK367793	BAJ98996	228	barke_contig_1823961 ^f^
*T. aestivum*	VPS60a	AK332171	–	232	nd
	VPS60b	tplb0015n24 ^c^	–	228	nd
*O. sativa*	VPS60a	NM_001060924	NP_001054389	232	Os05g0102900
	VPS60b	NM_001185188	NP_001172117	232	Os01g0102950
*Z. mays*	CHMP5a	NM_001157823	NP_001151295	228	GRMZM2G092468
	CHMP5b	BT040588	ACF85593	230	GRMZM2G069827
*A. thaliana*	VPS60.1	NM_111900	NP_187675	235	At3g10640
	VPS60.2	NM_120567	NP_568143	235	At5g04850

Footnotes explain database identifiers other than those of GenBank. Cereal ESCRT-III subgroup member designation was chosen to follow *A. thaliana* nomenclature with numbering for VPS2 in cases of cereal members grouping together with the corresponding *A. thaliana* member. Alphabetic characters were used for cereal members where this is not the case (i.e. SNF7a, b, c). *Arabidopsis* members have been annotated in (Winter and Hauser 2006)

^a^ EST contig identifier of KOMUGI/Wheat genetic Resources database database

^b^ DFCI *Oryza sativa* Gene Index (OsGI): http://compbio.dfci.harvard.edu/cgi-bin/tgi/tc_report.pl?tc=TC516772&species=rice

^c^ cDNA identifier of KOMUGI/Wheat genetic Resources database database

^d^ DFCI *Zea mays* Gene Index (ZmGI): http://compbio.dfci.harvard.edu/cgi-bin/tgi/tc_report.pl?species=maize&tc=TC482666

^e^ B73 RefGen_v3 Scaffold 340, incomplete

^f^ Genomic identifier does not cover full length of transcript

^g^ Genomic identifiers of barley are based on *Hordeum vulgare* Morex genome

^h^ cDNA/protein sequences are from *Hordeum vulgare* Haruna Nijo

^i^ 5′ region of cDNA is not aligned to genomic identifier

### Constructs and molecular biology

PMA-EGFP (PE) and PMA-EGFP-UB (PEU) reporter constructs were a gift of Swen Schellmann (Herberth et al. 2012).

#### p*Actin*::VPSx-XFP constructs

Coding sequences of HvVPS24, HvSNF7a and HvVPS60a were synthesized based on *H. vulgare* Haruna nijo AK366876, AK376941 and AK372130, respectively, and cloned into pBluescript II SK(+) by GeneCust. HvVPS24 and HvSNF7a.1 were synthesized as N-terminal fusions to mCherry (AAV52164.1 (Shaner et al. 2004)) and mEosFP (AAV54099 with V123T and T158H for retaining monomeric state (Wiedenmann et al. 2004)), respectively, and inserting an *Xmn*I restriction site between HvVPSx and XFP sequences. Nucleotide sequences of mCherry and mEosFP were codon optimized for barley using GENEius (Eurofins).

To obtain HvVPS24-mEos driven by the rice *actin1* promoter, first, HvVPS24-mCherry was transferred via *Sal*I/*Hind*III into the binary vector pSB277 (Kapusi et al. 2013) thereby replacing gfpS65T, followed by exchanging mCherry with mEosFP using restriction sites *Xmn*I/*Asc*I. To obtain *actin*::HvSNF7a-mEosFP, HvVPS24-mCherry was replaced with HvSNF7a-mEosFP using *Xmn*I/*Asc*I. *actin*::mEosFP was derived from *actin*::HvSNF7a-mEosFP by deletion of HvSNF7a with *Swa*I/*Xmn*I. To obtain *actin*::HvVPS60a-mCherry, gfpS65T in pSB277 was replaced via *Sal*I/*Hind*III with HvVPS60a-mCherry.

### Transient transformation of barley endosperm cells

Palea, lemma and dorsal pericarp tissue were removed with forceps from 12 to 15 dap barley caryopses to dissect the aleurone layer. For bombarding subaleurone cells, additionally, the aleurone layer was removed with forceps. Caryopses were placed on one half MS medium supplemented with 1 % sucrose and 3 gl^−1^ phytagel. The Biolistic® PDS-1000/He Particle Delivery System (BIO-RAD) was used for transient transformation. As described by Sanford et al. 1993, 0.6 μm gold particles were washed and coated. Bombardment was performed twice with 6 μl of coated particles per shot, at 27-in. Hg chamber vaccum, 900 psi. Distances from rupture disc to macrocarrier and macrocarrier to stopping screen were approximately 2 and 1 cm, respectively. The target shelf was inserted at position 3 from below.

### Confocal microscopy

Sections of transiently transformed seeds were mounted in tap water within a small border of vaseline to avoid sample floating, and glue was used to stabilize the cover slips. Images were captured by sequential scan using the Leica SP5 confocal laser scanning microscope with the following filter settings: mEosFP, excitation wavelength 488 nm, emission wavelength 508–540 nm; for mCherry, excitation 514, emission 580–640 nm; and for GFP, excitation wavelength 488 nm, emission wavelength: 503–530 nm. Images were processed using Leica confocal software version 2.61, ImageJ and Adobe Photoshop CS5.

### Statistical analyses

The mean values of the percentage values were calculated for each biological replicate and the standard deviation (sd) was calculated. The sd should be neglected in cases where only one biological replication exists. We chose a two-sample t-test to calculate the significance as following: t-test was calculated from the percentage values of comparable, different groups that have unequal variances. Data are significant when *P* < 0.05.

## Results

### Annotation of ESCRT-III members expressed in cereals

**Fig. 1:**
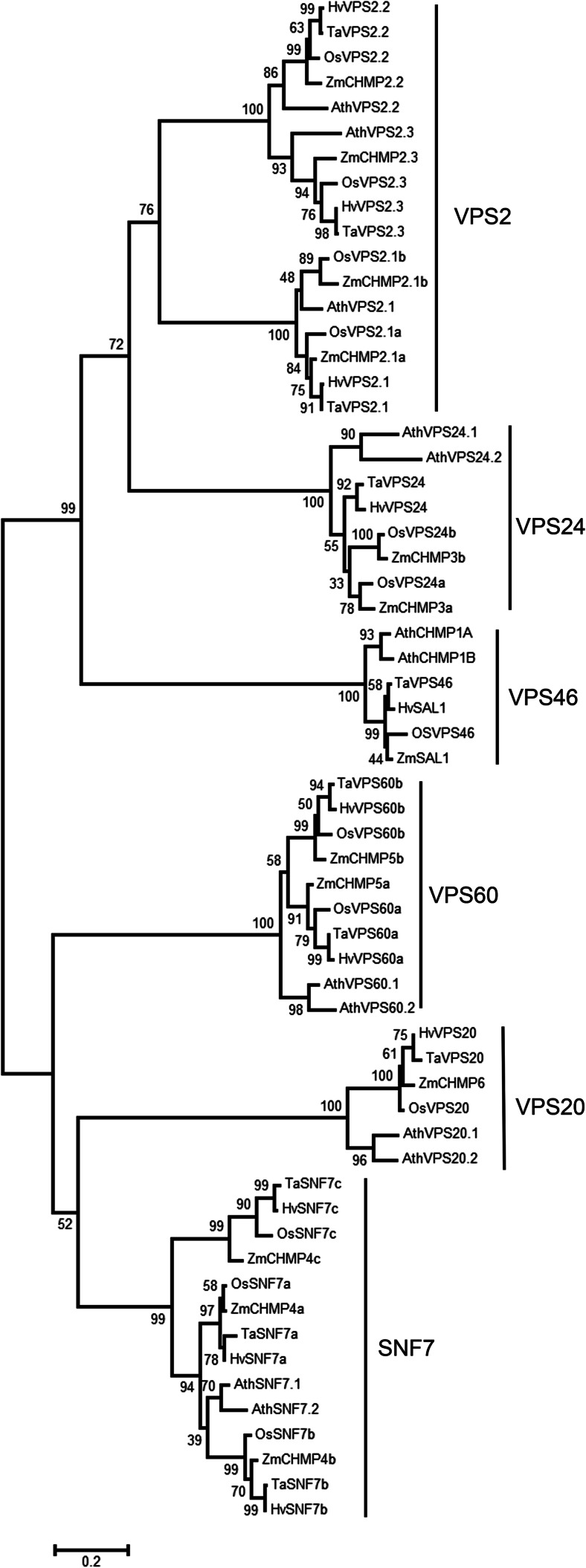
Neighbour joining (*NJ*) tree of 61 protein sequences of ESCRT-III members of *A. thaliana* and barley, wheat, maize and rice presented in Table 1 and based on the alignment provided in Supplemental Figure 1. The NJ tree was built using standard configurations in MEGA6 (Tamura et al. 2013). Distances using the Poisson correction method are given in the units of the number of amino acid substitutions per site. All positions containing gaps were eliminated. Bootstrap values (1000 replicates) are shown at the branches

Functional knowledge and the annotation of ESCRT-III components in cereals are missing—with the exception of SAL1 in maize and barley (Olsen et al. 2008; Shen et al. 2003). ESCRT-III members constitute a family of small coiled coil proteins which are divided into the two subfamilies SNF7-VPS20-VPS60 and VPS2-VPS24-VPS46; the number of paralogs for each subunit member varies between organisms (Howard et al. 2001; Leung et al. 2008; Winter and Hauser 2006). To build an inventory of ESCRT-III members in cereals, known *A. thaliana* proteins (Winter and Hauser 2006) were used to search public databases for homologs with evidence for full-length expressed sequences (cDNAs or EST contigs) in barley, wheat, rice and maize. A NJ tree including *A. thaliana* proteins was used to allocate newly identified members to ESCRT-III subunits (Fig. 1). The corresponding alignment can be found as Supplementary Material 1; a list with database identifiers is provided in Table 1.

Components with evidence for expression were detected for all ESCRT-III subunits in barley, wheat, maize and rice (Table 1, Fig. 1). In each of these species, one homolog was detected for VPS2.2, VPS2.3, VPS20 and VPS46. Two and three homologs were identified for VPS60 and SNF7, respectively. Barley and wheat possess one and maize and rice two homologs of VPS2.1 and VPS24. On the NJ tree, each of the two *Arabidopsis* paralogs for VPS20, VPS24, VPS46 and VPS60 is found as separate clades to cereal members on respective subtrees. AtVPS2.1, AtVPS2.2 and AtVPS2.3 are placed within monophyletic groups with the respective cereal members. The *A. thaliana* paralogs SNF7.1 and SNF7.2 are placed with high bootstrap support, yet as separate clade, with cereal SNF7a and SNF7b.

### Expression analysis of ESCRT-III in barley endosperm

Mass spectrometry was previously used to construct an atlas of developing maize seed proteotypes comprising approximately 14,000 proteins extracted from embryo, endosperm and aleurone/pericarp fractions at seven stages of development (Walley et al. 2013). We used the atlas to search for ESCRT-III members. Normalized and averaged data show that members of all ESCRT-III subunits are expressed during development in endosperm and in aleurone/pericarp tissue in maize (Table S2). Based on the Affymetrix Barley1 Gene Chip, expression data of grain maturation, desiccation and germination of two tissue fractions (starchy endosperm/aleurone and embryo/scutellum) are available for barley (Sreenivasulu et al. 2008). They indicate that homologs to *Arabidopsis* ESCRT-III (HvVPS2.1, HvSAL1, HvSNF7a/c, HvVPS24, HvVPS60a/b) are expressed during analysed stages of grain development and germination. Furthermore, homologs to AtVPS28 (ESCRT-I) and AtVPS36 (ESCRT-II) are active (Table S3).

**Fig. 2:**
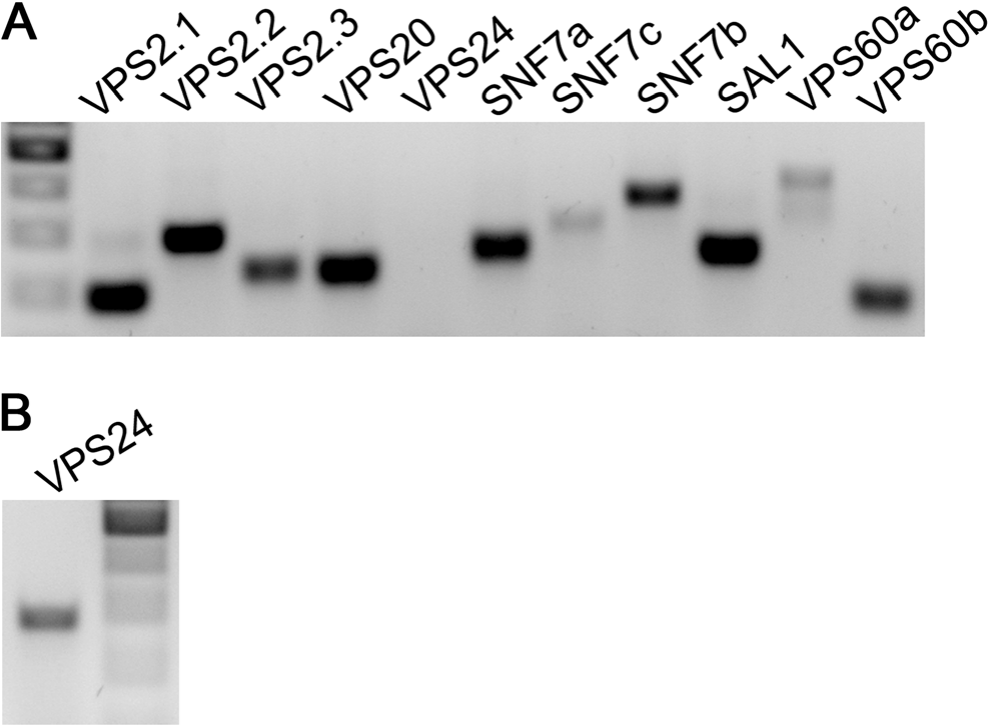
ESCRT-III members are expressed in barley endosperm. Gene-specific primers were used to detect expression of ESCRT-III members in barley endosperm cDNA (10–12 dap). **a** PCR on 1:10 diluted cDNA of barely endosperm. **b** PCR for VPS24 on undiluted cDNA of barley endosperm. DNA marker 500–200 bp

To establish whether all detected ESCRT-III family members are expressed in barley endosperm, real-time PCR (RT-PCR) was performed using RNA isolated from endosperm tissue (including starchy endosperm and aleurone) of 12-dap-old caryopses. All ESCRT-III members of barley are expressed in endosperm tissue (Fig. 2a, b) supporting a possible functional role of ESCRT in endosperm development, in line with sal1 and vps22 mutants in maize and rice, respectively (Shen et al. 2003; Zhang et al. 2013).

### ESCRT-III members are distributed in the cytosol, at agglomerations and at the plasma membrane in barley endosperm

**Fig. 3:**
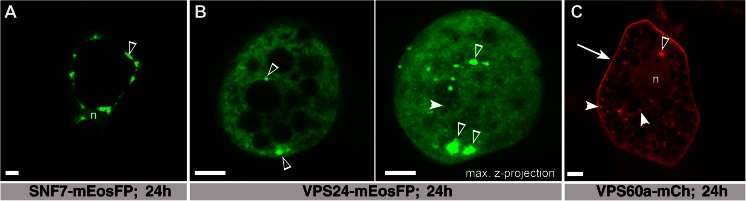
Localization of HvSNF7a, HvVPS24 and HvVPS60a in barley endosperm. **a** *Actin*::HvSNF7a-mEosFP induces large agglomerations (*open arrowhead*) within a cell in the embryo surrounding region. Note the weak signal in the nucleus (*n*). **b** *Actin*::HvVPS24-mEosFP localizes at vesicles (*arrowhead*) and to the cytoplasm concomitant with inducing agglomerations (*open arrowheads*). Note the maximal z-projection of 16 1-μm sections. **c** *Actin*::HvVPS60a-mCh localizes at the PM (*arrow*), vesicles (*arrowhead*), agglomerations (*open arrowhead*) and to a minor extent in the nucleus (*n*). Confocal single scans were made 24 h after bombardment. *Scale* = 5 μm

To study the subcellular localization of ESCRT-III, barley ESCRT-III members SNF7a, VPS24 and VPS60a were chosen for transient expression studies by particle bombardment of barley endosperm. The genes were placed under the control of the rice *actin*1 promoter (McElroy et al. 1991) and fluorescently tagged at their C-terminus with fluorescent proteins mEos and mCherry, which have been chosen for their ability to retain a monomeric state (see “Materials and methods”). C-terminally tagged plant ESCRT-III members AtVPS2.1 and AtVPS2.2 exhibited dominant negative effects (Ibl et al. 2012; Katsiarimpa et al. 2013) probably because of impaired auto-inhibition as analysed for C-terminally tagged yeast and mammalian ESCRT-III proteins (Teis et al. 2008; Zamborlini et al. 2006). Auto-inhibition is regulated via closed and open conformation of the C-terminal alpha-helix and ensures regulation of assembly and disassembly of the ESCRT-III complex at membranes (Henne et al. 2012). Release of auto-inhibition leads to enhanced accumulation on endosomes, termed class E compartments in yeast (Teis et al. 2008). The experimental setup therefore allowed us to analyse the distribution and behaviour of ESCRT-III members in cells with superimposed recombinant ESCRT-III load. After 24 h, barley endosperm was screened for expression by in vivo (fluorescence and) confocal microscopy, revealing strong fluorescent signals in barley endosperm. In addition to very weak signals in the cytosol and in the nucleus, a strong signal of HvSNF7a-mEosFP could be detected at agglomerations of different sizes (Fig. 3a). HvVPS24-mEosFP localizes in the cytosol and at agglomerations in aleurone cells, highlighted by a maximal z-projection (Fig. 3b). HvVPS60a-mCh could be visualized strongly at the plasma membrane, at agglomerations and in the cytosol (Fig. 3c). Contrary, a maximal z-projection of *actin*::mEosFP showed a weak signal in the cytosol and in the nucleus, confirming the authenticity of the signals from the barley ESCRT-III fusion proteins (Suppl. Fig. 1). In summary, confocal microscopy studies showed that the recombinant barley ESCRT-III members HvSNF7a, HvVPS24 and HvVPS60a are localized in the cytosol and at agglomerations and HvVPS60a additionally at the plasma membrane in barley endosperm.

### Localization of HvVPS60a at the plasma membrane in aleurone versus association with PSV membranes in subaleurone

**Fig. 4:**
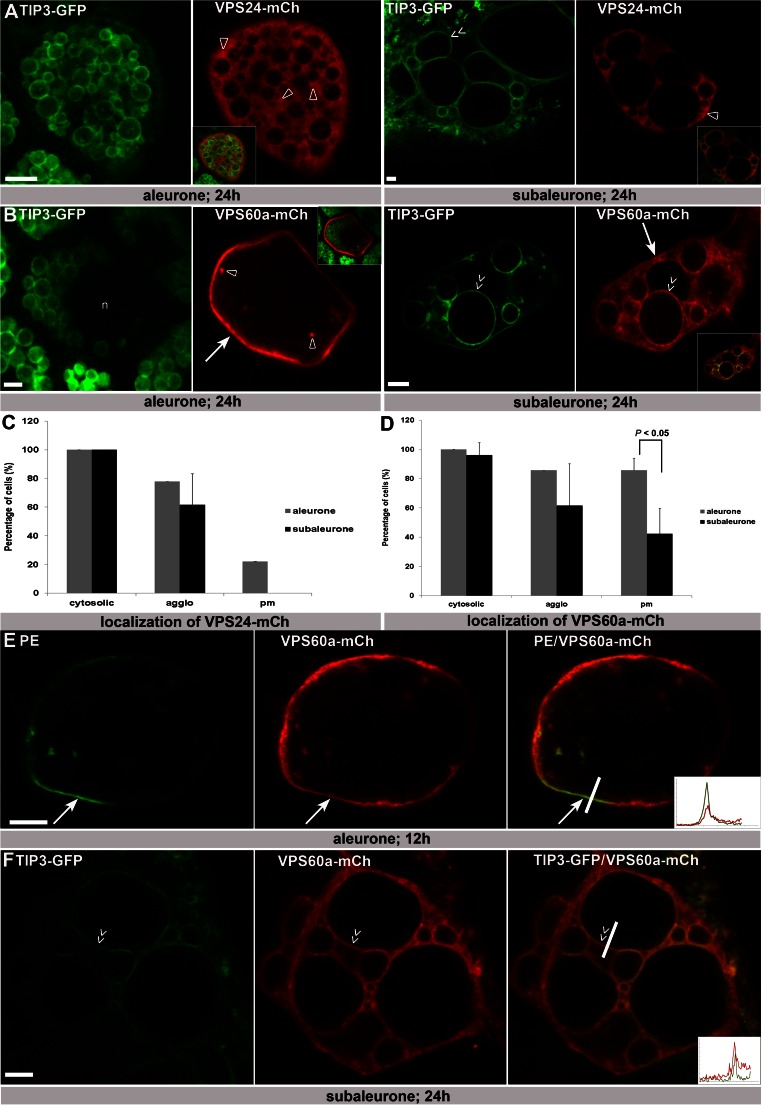
The localization of HvVPS60a-mCh differs in aleurone and subaleurone and shows plasma membrane localization in aleurone. **a** Single scans of barley aleurone and subaleurone cells showing transient transformation of HvVPS24-mCh in TIP3-GFP lines. In both, aleurone and subaleurone, HvVPS24-mCh localizes cytosolic and at small agglomerations (*open arrowheads*). Note the close ups to visualize co-localization. **b** Confocal single scans of HvVPS60a-mCh in TIP3-GFP lines and of co-transformation of HvVPS60a-mCh with TIP3-GFP. In aleurone, HvVPS60a-mCh shows strong signal at the pm (*arrow*), weak in the cytosol in parallel to small agglomeration structures (*open arrowheads*). Note the altered localization of HvVPS60a-mCh in subaleurone cells. HvVPS60a-mCh signal could be detected close to heterogenous, spherical PSVs (*double arrow*) but reduced at the PM (*arrow*). Note the close ups to visualize co-localization. **c**, **d** Localization of HvVPS24-mCh and HvVPS60a-mCh in aleurone and subaleurone. Relative number of cells showing localization in cytosol at agglomerations and at the PM was scored. Total number of cells: HvVPS24-mCh in aleurone (biological replicates 1; *n* = 18) and in subaleurone (biological replicates 3; *n* = 13); HvVPS60a-mCh in aleurone (biological replicates 1; *n* = 14) and in subaleurone (biological replicates 3; *n* = 24). The difference in the localization of HvVPS60a-mCh at the PM in aleurone and subaleurone is statistically significant (*P* < 0.05). Plus ends of the standard deviation are indicated. **e** Single scan of an aleurone cell showing co-localization of the plasma membrane marker PMA-EGFP (PE) and HvVPS60a-mCh at the pm (*arrow*). Fluorescence profile: *X*-axis, distance [μm] from 0 to 6 μm; *Y*-axis, intensity from 0 to 100. **f** Single confocal scan of a subaleurone cell that shows co-localization of HvVPS60a-mCh and the vacuolar membrane signal of the TIP3-GFP line. Note the *double arrows* indicating co-localization of HvVPS60a-mCh with the PSV membrane. Fluorescence profile: *X*-axis, distance [μm] from 0 to 8 μm; *Y*-axis, intensity from 0 to 100. Confocal single scans and *z*-series were made 24 h after bombardment. *Scale* = 5 μm

Recent data show the distinct behaviour of the endomembrane system in barley aleurone, subaleurone and starchy endosperm during seed development (Ibl et al. 2014). While the morphology of protein storage vacuoles (PSVs) remains unchanged in aleurone cells, PSVs in subaleurone and starchy endosperm are extremely dynamic, reflecting a constantly remodelling endomembrane system in these layers. To test if the subcellular distribution of ESCRT-III differs between cell layers, HvVPS24-mCh and HvVPS60a-mCh were transiently expressed in OsTIP3::TIP3-GFP lines (Ibl et al. 2014) to specifically study the localization of HvVPS24-mCh and HvVPS60a-mCh in both aleurone and in subaleurone. In aleurone, 18 positively transformed cells (biological replicates 1) show that HvVPS24-mCh localizes in all cells diffusely in the cytoplasm, additionally in 78 % of cells at agglomerations and 22 % at the plasma membrane (Fig. 4a, c). However, the intensity of the plasma membrane signal is not increased compared to the cytosolic signal (Suppl. Fig. 2). In subaleurone, HvVPS24-mCh localizes in 13 cells (biological replicates 3) to 100 % cytosolic and to 62 % at agglomerations, showing that the localization of HvVPS24-mCh is similar in aleurone and subaleurone cells (Fig. 4a). Moreover, HvVPS24-mCh is not associated to PSV membranes (Fig. 4a, subaleurone, double arrows). Contrary, the distribution of HvVPS60a-mCh differs between aleurone and subaleurone (Fig. 4b, d). In aleurone, confocal microscopy studies (biological replicates 1, *n* = 14) revealed a weak signal for HvVPS60a-mCh in the cytosol (100 % of cells), at cytoplasmic agglomerations (86 % of cells) (Fig. 4a, asterisks) and strongly at the plasma membrane (100 % of cells) (Fig. 4b, arrow). To investigate HvVPS60a-mCh localization at the plasma membrane further, we used the Arabidopsis integral plasma membrane ATPase (PMA-EGFP; PE; (Herberth et al. 2012)) as a marker (Suppl. Fig. 3). Confocal single scans of co-bombarded cells reveal co-localization at the plasma membrane (Fig. 4e). In subaleurone, we observed (biological replicates 5, *n* = 26) a strong cytosolic signal (96 % of cells), fewer agglomerations (62 % of cells) and a reduced number of cells with signal at the plasma membrane (42 % of cells). Moreover, a possible association with TIP3-GFP-labelled PSV membranes could be observed in 50 % of the cells (Fig. 4b, double arrow). In detail, a TIP3-GFP-labelled PSV membrane shows strong labelling of HvVPS60-mCh (Fig. 4f, double arrow), indicating co-localization at the tonoplast. Altogether, these data may indicate a spatially regulated localization pattern of HvVPS60a-mCh in barley aleurone.

### Re-localization of HvVPS60a and HvVPS24 to large agglomerations in both aleurone and subaleurone

**Fig 5:**
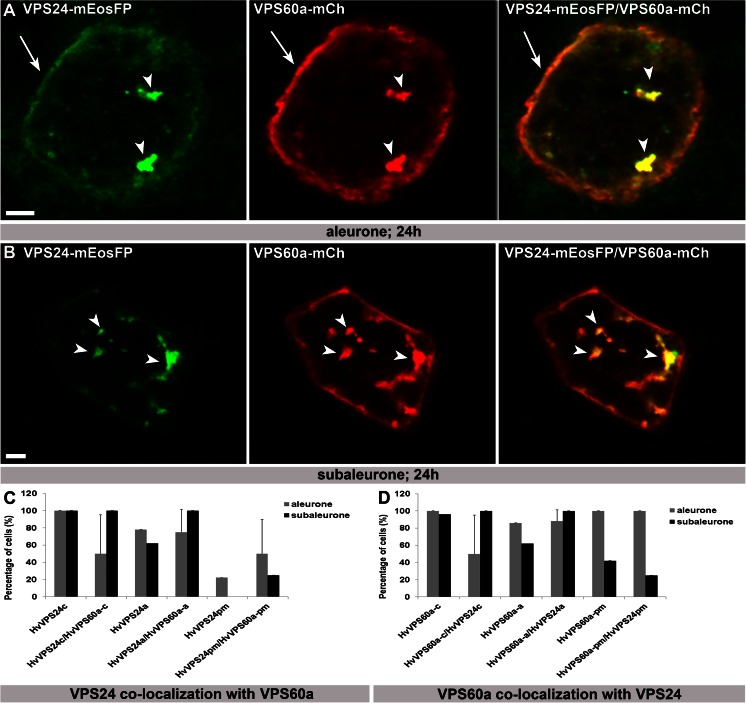
Co-bombardment of HvVPS24-mEosFP with HvVPS60a-mCh induces re-localization of both proteins to large agglomerations in aleurone and subaleurone. **a**, **b** HvVPS24m-Ch and HvVPS60a-mCh are re-localized from cytoplasm to large globulare structures (*arrowheads*) in aleurone and subaleurone. Note the additional localization of VPS24m-Ch and VPS60a-mCh at the PM in aleurone (*arrows*). **c**, **d** 8 and 4 co-transformed cells within 5 and 1 biological replicates, respectively, were analysed in aleurone and subaleurone. Relative number of cells showing localization in cytosol (-*c*), at agglomerations (-*a*) and at the plasma membrane (-*pm*) was scored and compared with single transformation. Plus ends of the standard deviation are indicated. Confocal single scans were made 24 h after bombardment. *Scale* = 5 μm.

As it is known that components of ESCRT complexes can be re-localized as a consequence of their interaction, we subsequently co-transformed the two putative interaction partners (Richardson et al. 2011) HvVPS60a-mCh and VPS24-mEosFP into both barley aleurone and subaleurone. Confocal microscopy analyses of co-transformed cells (aleurone *n* = 8; subaleurone *n* = 4; biological replicates aleurone/subaleurone 5/1) reveal re-localization of both HvVPS60a-mCh and HvVPS24-mEosFP to large, globular structures (Fig. 5a, b, arrowheads). In detail, previous nearly 100 % cytosolic localization of both HvVPS24 and HvVPS60a in aleurone is reduced to 50 % (Fig. 5c, d), whereas the accumulation of HvVPS24 at the plasma membrane increased from 20 % of singly transformed cells to 50 % of co-bombarded cells in aleurone and to approximately 30 % in subaleurone (Fig. 5c). These in vivo microscopic data suggest that the localization of transiently expressed ESCRT-III proteins is influenced by simultaneous overexpression of putative interaction partners.

### HvVPS60a inhibits vacuolar sorting of a monoubiquitinated plasma membrane protein in aleurone but not in subaleurone

**Fig. 6:**
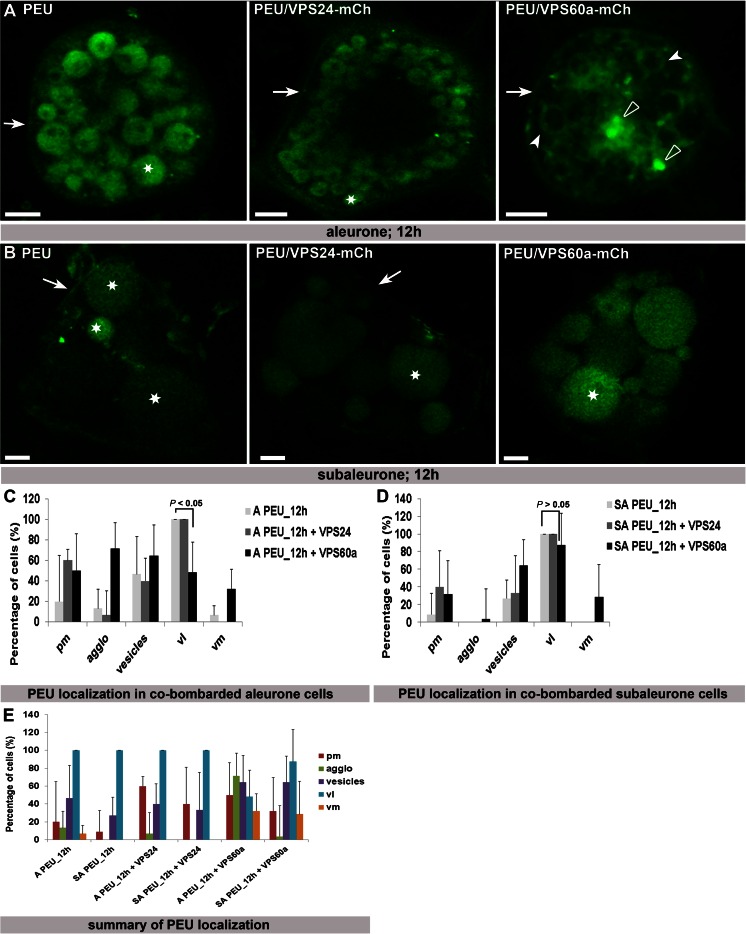
Localization of PE and PEU in co-transformed aleurone and subaleurone cells. **a** PEU was sorted in the vacuole (*asterisk*) in aleurone cells. PEU is located at the PM (*arrow*) and sorted in vacuoles (*asterisk*) in co-bombarded HvVPS24-mCh cells. Note the affected sorting of PEU in co-transformed HvVPS60a-mCh cells, where PEU is localized at the pm (*arrow*), at agglomerations (*open arrowheads*) and at vacuolar membranes (*arrowheads*). **b** PEU is localized at the pm (*arrowhead*) and sorted in the vacuole in subaleurone cells (*asterisks*). In HvVPS24-mCh co-bombarded cells, PEU is localized weakly at the pm (*arrowhead*) and sorted in the vacuole. PEU is also sorted in the vacuole in co-transformed HvVPS60a-mCh cells (*asterisk*). *Scale* = 5 μm. **c** Statistical analyses of localizations of PEU in co-transformed HvVPS24-mCh and HvVPS60a-mCh aleurone cells. Biological replicates: 3 and 5 times for co-transformation with HvVPS24m-Ch and HvVPS60a-mCh, respectively; *n* (co-transformed cells with HvVPS24-mCh/HvVPS60a-mCh: 15/28). **d** Statistical analyses of localization of PEU in co-transformed HvVPS24-mCh and HvVPS60a-mCh aleurone cells. Biological replicates: 3 and 6 times for co-transformation with HvVPS24m-Ch and HvVPS60a-mCh, respectively; *n* (co-transformed cells with HvVPS24-mCh/HvVPS60a-mCh: 15/28). The difference in the localization of PEU in aleurone cells in the vacuolar lumen alone or in the presence of HvVPS60a-mCh is statistically significant (*P* < 0.05), whereas in subaleurone, the difference in the localization of PEU alone or in presence of HvVPS60a-mCh is not significant (*P* > 0.05). Plus ends of the standard deviation are indicated. **e** Summary of the statistical analyses of PEU in co-transformed HvVPS24m-Ch and HvVPS60a-mCh aleurone and subaleurone cells. Note the different localization of PEU in co-transformed HvVPS60a-mCh aleurone cells. Plus ends of the standard deviation are indicated. *A* aleurone, *SA* subaleurone, *pm* plasma membrane, *agglo* agglomeration, *vl* vacuolar lumen, *vm* vacuolar membrane.

To investigate whether overexpressed HvVPS24 or HvVPS60a have an inhibitory effect on vacuolar sorting of plasma membrane protein, the endocytosis of an artificial MVB cargo PMA-EGFP-UB (PEU) was investigated in aleurone and subaleurone cells after co-transformation with HvVPS24m-Ch or HvVPS60a-mCh. Monoubiquitination of the Arabidopsis plasma membrane ATPase PMA-EGFP (PE) induces the endocytosis and sorting into the vacuolar lumen (Herberth et al. 2012). As previously reported, co-expression of both, a dominant negative version of the Arabidopsis AAA-ATPase AtSKD1(EQ) that is required for the disassembly of ESCRT-III and the ESCRT-III component VPS2.1 fused to a C-terminal TagRFP, have an inhibitory effect on the sorting of PEU via the MVB pathway towards the vacuole (Herberth et al. 2012; Katsiarimpa et al. 2013). We transiently transformed PE and PEU in aleurone and subaleurone cells and observed PE at the pm and PEU in the vacuolar lumen within 12 h after transformation (Suppl. Fig. 3). Twelve hours after co-bombardment of PEU with HvVPS24-mCh in aleurone, we could observe PEU at the pm (Fig. 6a, arrow) and in the vacuolar lumen (Fig. 6a, asterisk), similar to the localization of PEU alone (Fig. 6a, c). When we analysed PEU in co-transformed HvVPS60a-mCh aleurone cells, PEU was detected at the plasma membrane (Fig. 6a, arrow), at agglomerations (Fig. 6a, open arrowhead), at the vacuolar membrane (Fig. 6a, arrowheads) and approximately in 40 % of the cells in the vacuolar lumen (Fig. 6c). Interestingly, analyses of HvVPS24-mCh and HvVPS60a-mCh in co-bombarded PEU subaleurone cells show localization of PEU at the plasma membrane (Fig. 6b, arrows) and in the vacuolar lumen (Fig. 6b, asterisks). As we could not detect differences in the qualitative fluorescence signal in the vacuolar lumen between PEU single transformation and PEU together with HvVPS60a-mCh, we conclude that HvVPS60a-mCh has hardly any inhibitory effect on the sorting of monoubuquitinated pm ATPase in subaleurone (Fig. 6b arrow, asterisks). Contrary to the PEU signal at the vacuolar membrane in co-bombarded HvVPS60a-mCh aleurone cells, where PEU was only detected at the vacuolar membrane but within the same cell not additionally in the vacuolar lumen, the PEU signal at the vacuolar membrane in co-bombarded HvVPS60a-mCh subaleurone cells could be detected at the vacuolar membrane concomitant with the signal in the vacuolar lumen (Fig. 6d). Thus, the sorting of PEU in HvVPS60a-mCh co-bombarded subaleurone cells is still provided (Fig. 6e). Together, these results indicate that HvVPS60a-mCh is involved in the vacuolar lumen sorting of monoubiquinated plasma membrane cargo in aleurone but not in subaleurone.

## Discussion

### ESCRT-III is expressed in developing barley endosperm

ESCRT in plants is traditionally studied in *Arabidopsis*, conserved and distinct organization to fungi and metazoan cells is being established (Korbei et al. 2013; Reyes et al. 2011a) and recently, an assay for screening ESCRT target candidates has been proposed (Cai et al. 2014). There are two lines of evidence favouring the transfer of ESCRT knowledge to cereals and especially to cereal endosperm (1) the sal1 mutant in maize with its developmental phenotype of an abnormal high number of aleurone layers (Shen et al. 2003) and (2) observations in seeds that implicate the MVB in trafficking of SSPs (and possibly proteases) to the PSV (Reyes et al. 2011b, reviewed in Ibl and Stoger 2012). Here, a comparative basic inventory of evidently transcribed ESCRT-III subunits in barley, wheat, maize and rice has been compiled by searching public databases. Expression of ESCRT components in endosperm tissues in *Arabidopsis* and barley is covered by microarray data (Richardson and Mullen 2011; Sreenivasulu et al. 2008) and in maize by mass spectrometry (Walley et al. 2013). In barley, we confirm by RT-PCR that all barley members in the annotated list are expressed in developing endosperm, including the paralogs of ESCRT-III members which are not present on the Affymetrix Barley1 GeneChip (HvVPS2.2, HvVPS2.3, HvSNF7c).

### Recombinant expression studies of ESCRT-III proteins

Localization studies of ESCRT components are rare in seeds and even more so in cereal endosperm. Static images of fixed samples by confocal and electron microscopy have been provided for SAL1 in maize endosperm (Tian et al. 2007), but to our knowledge, no in vivo studies are present due to the technical complexity of cereal endosperm. Here, we present in vivo localization studies of HvSNF7a, HvVPS24 and HvVPS60a in different layers of developing barley endosperm.

Within this work, we constructed and expressed ESCRT-III components C-terminally tagged with monomeric fluorophores (Shaner et al. 2004; Wiedenmann et al. 2004), i.e. versions with abolished intermolecular fluorophor interaction, and driven by the rice *actin*1 promoter (McElroy et al. 1991). Aleurone and subaleurone cells were transiently transformed by particle bombardment. C-terminally tagged ESCRT-III fusion proteins have been used to elucidate, for example, their membrane binding behaviour in mammalian cells or ESCRT-III complex assembly in yeast, and C-terminally tagged AtVPS2.2 has been shown to partially complement a knock-out mutant in *Arabidopsis* (Hanson et al. 2008; Ibl et al. 2012; Teis et al. 2008). ESCRT-III, in contrast to ESCRT-I and II, is not a stable complex. Its monomeric components are recruited to form complexes at membranes with particular stoichiometry, and during or after membrane scission, they again dissociate fuelled by the action of the AAA+ ATPase VPS4 (Henne et al. 2013). C-terminally tagged ESCRT-III components exhibiting dominant negative behaviour have been shown (Ibl et al. 2012; Katsiarimpa et al. 2014; Pawliczek and Crump 2009; Strack et al. 2003; Teis et al. 2008), and this is explained by impaired auto-inhibition to retain monomeric state. In our experimental setup, therefore, an enhanced accumulation (in comparison to endogenous levels) of ESCRT-III proteins at target membranes in the remodelling endomembrane system of barely endosperm might be attributed to both, distortion of ESCRT-III component stoichiometry by superimposed expression and by dominant negative effects due to C-terminal tagging. Furthermore, impaired dissociation of ESCRT-III complexes might lead to ectopic localization at and thus tagging of membranes further downstream in ESCRT pathways in which they are involved. Dominant negative effects in turn can be exploited to functionally test involvement of ESCRT-III components in protein sorting pathways in both cell layers.

### HvSNF7a and HvVPS24 induces large agglomerations in barley endosperm

Within this work, transiently overexpressed HvSNF7a-mEosFP localized at considerable agglomerations in barley endosperm. Recent *Arabidopsis* ESCRT protein interactome studies pointed out that Snf7A and Snf7B proteins are also capable of homotypic and heterotypic interactions, consistent with a conserved role for Snf7 oligomerization during ESCRT-III assembly in plants (Richardson and Mullen 2011; Shahriari et al. 2011). Richardson and Mullen (2011) further showed that the *Arabidopsis* Snf7A (i.e. HA-Snf7A) localized in BY-2 cells to punctate and large, globular like structures (Richardson and Mullen 2011). It was pointed out that these large globular structures may be also the result of the assembly of abnormal Snf7A oligomers as they were observed before in yeast and mammalian cells, respectively, to form large, higher-ordered polymers on the endosomal surface (Teis et al. 2010) and induce the formation of aberrant MVB-related structures upon their overexpression in cells (Peck et al. 2004; Boysen and Mitchell 2006). Thus, the large agglomerations induced by transiently expressed HvSNF7a-mEosFP point to a protein with intact membrane association capacity and they indicate a functional role of HvSNF7a in barley endosperm.

Similarly, overexpressed Vps24 was reported to become membrane-associated and to perturb endosomal structure in mammalian cells (Lin et al. 2005). In barley endosperm, transient overexpression of HvVPS24-mEosFP induced prominent agglomerations in addition to cytosolic localization even though these compartments were smaller compared to HvSNF7a-mEos FP-induced compartments. These results support the hypothesis that the relative stoichiometry of ESCRT-III subunits is an important factor in ESCRT-III assembly (Teis et al. 2008) and that the increased steady-state association of ESCRT-III proteins with membranes caused by overexpression is inducing agglomerations of different sizes, causing dysfunction in the MVB pathway (Lin et al. 2005).

### Particular localization of HvVPS60a at the plasma membrane in barley aleurone

Here, we present subcellular localization data of HvVPS60a in plants. Our microscopic analysis of transiently expressed HvVPS60a-mCh shows cytosolic localization together with a strong signal at small aggregates (~1 μm) and at the PM in barley aleurone.

Localization of ESCRT proteins at the PM has been reported in mammalian cells and plants (Ibl et al. 2012; Jimenez et al. 2014; Richardson et al. 2011). While the functional significance of the plasma membrane localization of ESCRT proteins is still unknown and needs to be established *in planta*, ESCRT components have been described in mammalian cells to be involved in plasma membrane repair (Jimenez et al. 2014) and in exosome secretion and composition (Colombo et al. 2013). As no single transformed HvVPS24 and HvSNF7a plasma membrane localization could be observed within this work in aleurone, we exclude that wounding by particle bombardment is responsible for the localization of HvVPS60a at the plasma membrane. Thus, we do not assume a specific role of HvVPS60a in plasma membrane repair. On the other hand, ESCRT-mediated secretion in plant cells has been discussed (Ibl et al. 2012), even though conclusive evidence is still missing. As it is known that membrane proteins dedicated for either lysosomal/vacuolar functions or exosomal release are sequestered into MVB intraluminal vesicles (ILV) (Katzmann et al. 2002; Piper and Katzmann 2007), we hypothesize that HvVPS60a is localized at MVBs that are finally fusing with the plasma membrane. As the recombinant HvVPS60a is possibly changing the steady-state assembly/disassembly of ESCRT-III, HvVPS60a may remain at the MVB and thus at the plasma membrane after fusion. It is worth to mention that HvVPS24 could only be detected in the aleurone at the plasma membrane in 20 % of cells compared to 80 % of cells with HvVPS60a, indicating that HvVPS60a is possibly additionally involved in a different MVB trafficking pathway. This hypothesis is confirmed by recent data where SAL1 could be detected to a subfraction of MVBs in Arabidopsis embryo cells (Tian et al. 2007). Interestingly, biochemical and morphological evidence has been provided that the distinct fates (endocytosis and exocytosis) rely on distinct populations of MVBs that coexist within the same mammalian cell (Buschow et al. 2009; Mobius et al. 2002; White et al. 2006; Wubbolts et al. 2003). MVBs for both protein delivery to PSVs and involved in secretion were described in maize endosperm (Costa et al. 2003; Reyes et al. 2011b) but to our knowledge, no data have been provided in plants of morphologically identical but biochemically different MVBs. Thus, co-localization studies with a MVB marker (e.g. FYVE marker) and HvVPS60a and inhibitory treatments (e.g. wortmannin) will offer information about diverse MVBs within the aleurone.

### Spatially regulated localization of HvVPS60a in barley endosperm

Transient transformation of HvVPS60a in transgenic barley OsTIP3::OsTIP3-GFP lines allowed us, first, to identify viable cells and, second, to differentiate aleurone from subaleurone cells. Previous work has shown that morphological changes of PSVs are cell layer dependent and particularly dynamic in subaleurone and starchy endosperm cells, consistent with a rapidly remodelling endomembrane system during development (Ibl et al. 2014). Our data point to a spatially regulated localization of HvVPS60a. As described, in aleurone, HvVPS60a is localized in the cytosol, at small agglomerations and at the plasma membrane, whereas in subaleurone, HvVPS60a is associated with PSV membranes.

Different localization of HvVPS60a in aleurone and subaleurone may reflect its varying function in protein sorting. In this study, we showed that the artificial MVB cargo PMA-EGFP-UB (PEU) is endocytosed and delivered into the vacuole in barley endosperm. Since overexpressed HvVPS60a is associated with PSV membranes in subaleurone, we hypothesized that in subaleurone cells, a defective steady-state membrane association of HvVPS60a is induced by the different remodelling endomembrane system and possibly affecting protein targeting to the vacuole (Ibl et al. 2014). Recently, mis-localization of dominant negative ESCRT-III mutants to the tonoplast was described suggesting that the mutants were defective in membrane dissociation resulting in enhanced membrane association (Cai et al. 2014).

The necessity of the ESCRT-III steady-state membrane association is supported by the induced re-localization of HvVPS24 to the plasma membrane in aleurone and subaleurone, indicating that both proteins are acting in the same pathway. Surprisingly, co-bombardment studies of HvVPS60a and HvVPS24 with PEU in aleurone and subaleurone showed that in the presence of HvVPS60a, PEU is inefficiently targeted to the vacuolar lumen in aleurone. Contrary, PEU could be detected in a higher percentage of cells in the vacuolar lumen in the subaleurone in the presence of HvVPS60a. Thus, we conclude that HvVPS60a has an inhibitory effect on targeting the monoubiquitinated plasma membrane protein in aleurone. In conclusion, our findings reinforce the concept of Richardson and Mullen (2011) that highlighted the very dynamic expression of *Arabidopsis* ESCRT across a wide range of organs, tissues and treatments possibly resulting in a complex interplay between the spatial and temporal regulation of the ESCRT machinery in *Arabidopsis*. Our observations suggest that the localization of HvVPS60a is spatially regulated in barley endosperm. Moreover, we show involvement of HvVPS60a in protein targeting of ubiquitinated protein towards the vacuole in aleurone. Our interpretation implies that the function of ESCRT-III depends on the cell layer in barley endosperm. These observations point out the necessity to study the ESCRT-III machinery at the molecular as well as at the cellular level in every single cell tissue, and it will be worth to include the temporal influence (developing stage) in the future studies to understand the spatial-temporal regulation of ESCRT-III in cereal endosperm. Transgenic ESCRT-III barley marker lines and ESCRT-III RNAi barley lines will pave the way to investigate the cell layer-specific steady-state assembly/disassembly of ESCRT-III and the function of ESCRT-III in barley endosperm.

## Electronic supplementary material

Below is the link to the electronic supplementary material.

Supplemental Figure 1 Background signal of *actin*::mEosFP in barley endosperm. The maximal z-projection of 33 0.5 μm sections shows a weak signal of *actin*::mEosFP in the cytoplasm and in the nucleus. Scale = 5 μm. (GIF 18 kb)

High Resolution Image (TIFF 80117 kb)

Supplemental Figure 2 Localization of HvVPS24-mCh at the plasma membrane. Confocal single scan of HvVPS24-mCh in TIP3-GFP aleurone cell showing that HvVPS24-mCh is weakly localized at the pm. Confocal single scans were made 24 h after bombardment. Scale = 5 μm. (GIF 109 kb)

High Resolution Image (TIFF 84855 kb)

Supplemental Figure 3 Localization of PE and PEU in aleurone and subaleurone. **a**, **b** Confocal single scan of PE and PEU in GP aleurone (a) and subaleurone (b). PE localizes at the pm (arrow) and PEU is internalized into the vacuolar lumen (asterisks) 12 h after bombardment. Scale = 5 μm. (GIF 90 kb)

High Resolution Image (TIFF 84223 kb)

Supplemental material 1 Amino-acid alignment of ESCRT-III members VPS2, VPS20, VPS24, SNF7, VPS46 and VPS60 of *A. thaliana*, barley, wheat, maize and rice (FAS 16 kb)

Supplemental Table 1 PCR primers used for expression analyses (DOCX 22 kb)

Supplemental Table 2 Maize ESCRT-III proteins present in endosperm filtered from Dataset 1, (Walley et al. 2013) (DOCX 20 kb)

Supplemental Table 3 Barley (*Hordeum vulgare* Barke) ESCRT-III genes expressed during development and germination in endosperm, filtered from Supplemental Table S2 of (Sreenivasulu et al. 2008) (DOCX 24 kb)
