# Supplementary figures and images for: Cell layer-specific distribution of transiently expressed barley ESCRT-III component HvVPS60 in developing barley endosperm
Source: Protoplasma. 2015 Mar 22;253(1):137–53. doi: 10.1007/s00709-015-0798-1 (PMC4712231; doi:10.1007/s00709-015-0798-1)

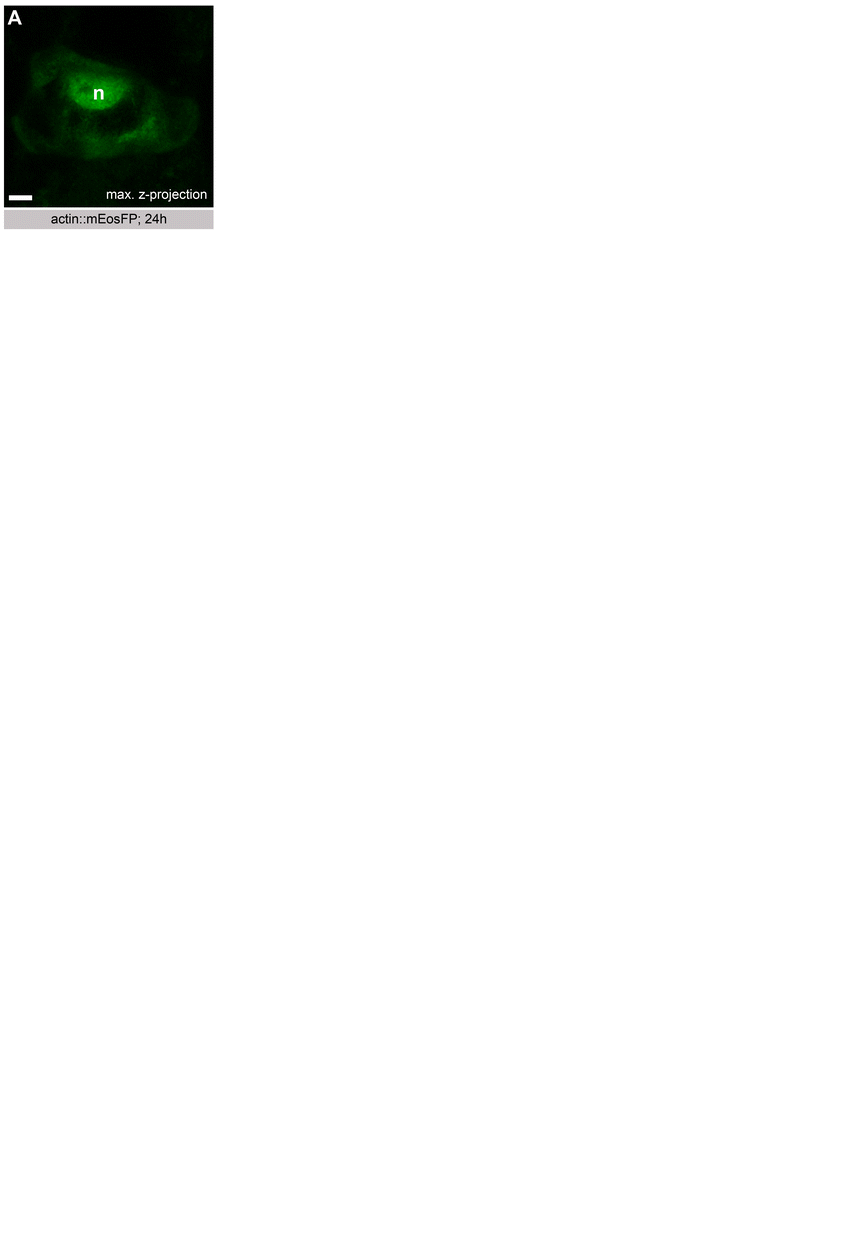

Supplement: Supplementary file 1 — Background signal of actin::mEosFP in barley endosperm. The maximal z-projection of 33 0.5 μm sections shows a weak signal of actin::mEosFP in the cytoplasm and in the nucleus. Scale = 5 μm. (GIF 18 kb) [file 709_2015_798_Fig7_ESM.gif]

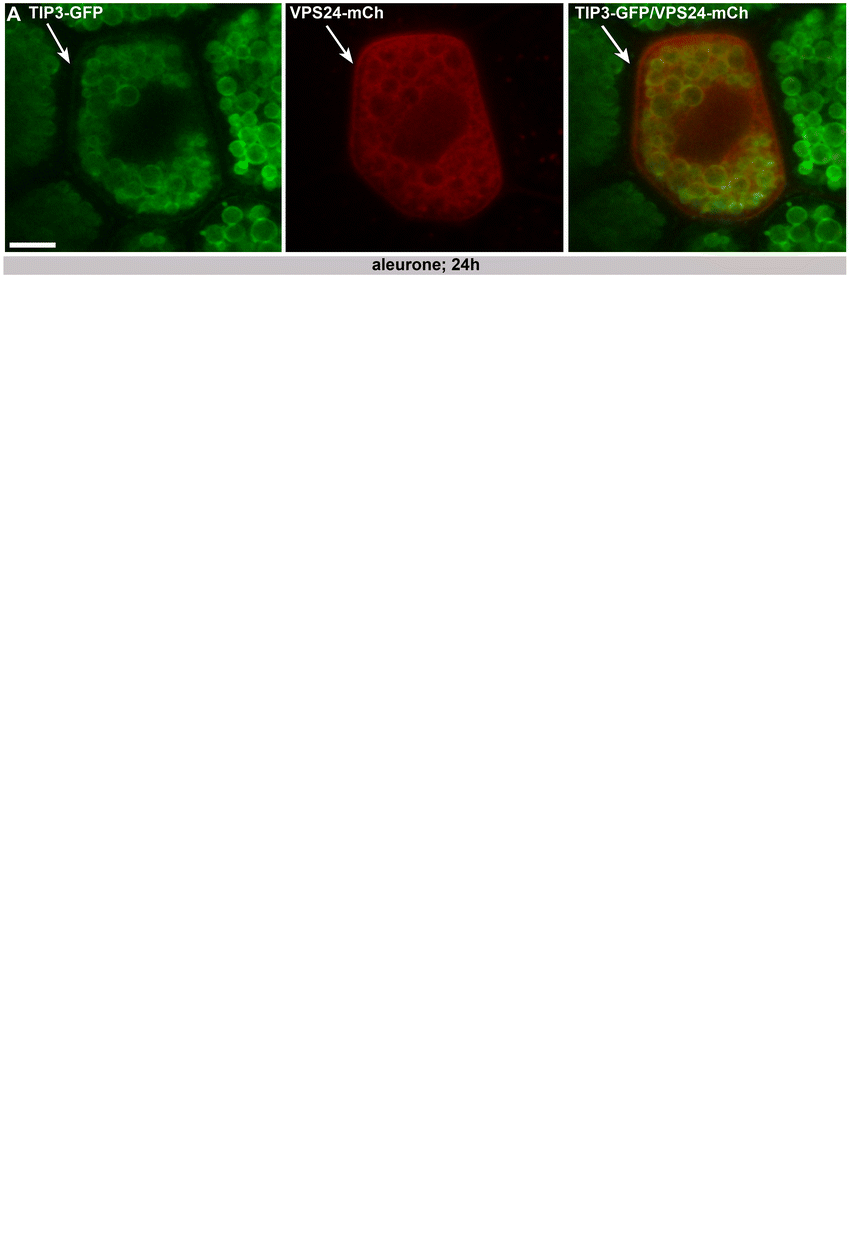

Supplement: Supplementary file 3 — Localization of HvVPS24-mCh at the plasma membrane. Confocal single scan of HvVPS24-mCh in TIP3-GFP aleurone cell showing that HvVPS24-mCh is weakly localized at the pm. Confocal single scans were made 24 h after bombardment. Scale = 5 μm. (GIF 109 kb) [file 709_2015_798_Fig8_ESM.gif]

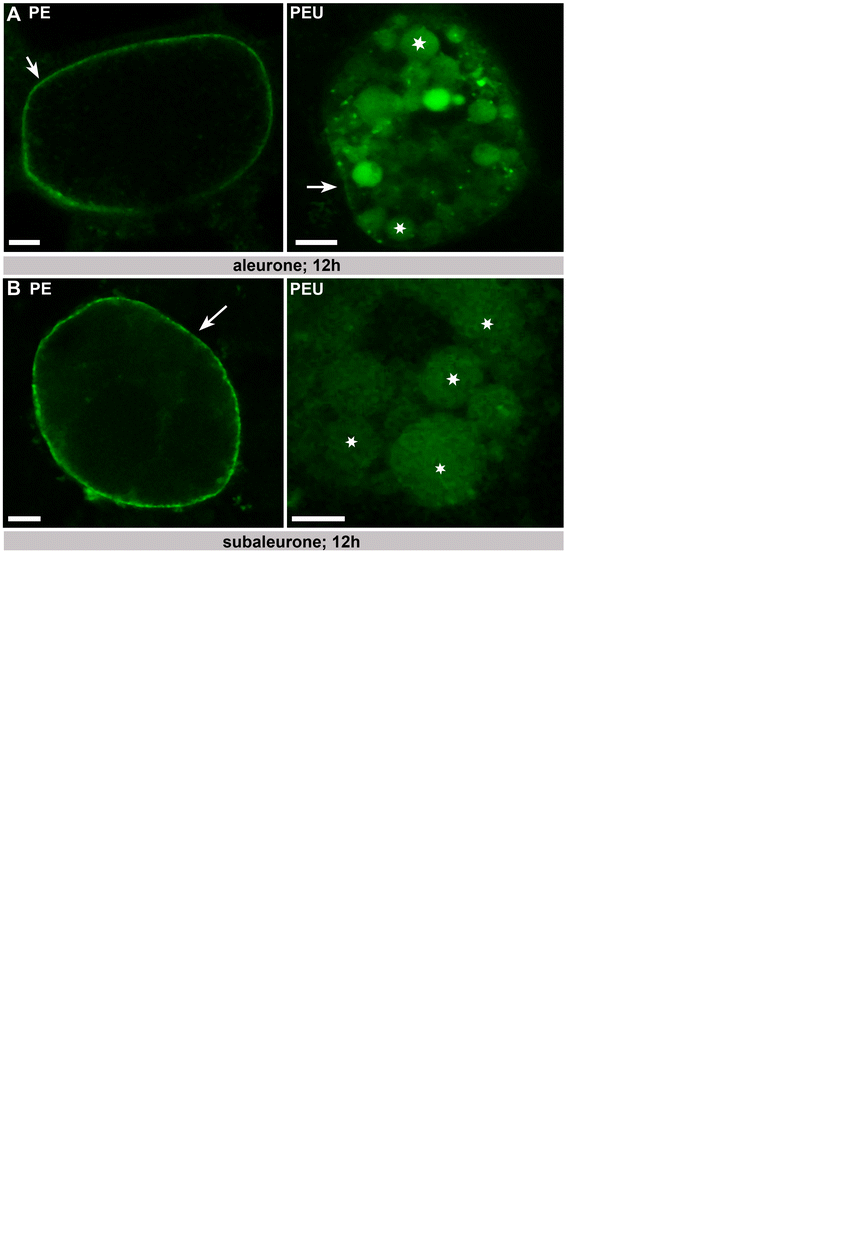

Supplement: Supplementary file 5 — Localization of PE and PEU in aleurone and subaleurone. a, b Confocal single scan of PE and PEU in GP aleurone (a) and subaleurone (b). PE localizes at the pm (arrow) and PEU is internalized into the vacuolar lumen (asterisks) 12 h after bombardment. Scale = 5 μm. (GIF 90 kb) [file 709_2015_798_Fig9_ESM.gif]
