# Supplementary material for: Cell layer-specific distribution of transiently expressed barley ESCRT-III component HvVPS60 in developing barley endosperm
Source: Protoplasma. 2015 Mar 22;253(1):137–53. doi: 10.1007/s00709-015-0798-1 (PMC4712231; doi:10.1007/s00709-015-0798-1)
Supplement: Supplementary file 8 — PCR primers used for expression analyses (DOCX 22 kb) [file 709_2015_798_MOESM5_ESM.docx]

Table S1 PCR primers

| Expression analysis | | | | |  | |
| --- | --- | --- | --- | --- | --- | --- |
| Gene | Direction | | | Sequence (5´- 3´) | Expected PCR product length * (b) | |
|  |  |  |  |  | cDNA | gDNA |
| VPS24 | | F | | GATGACAAAGGCTGGCGTAATG | 286 | 396* |
|  |  | R | | CGACCTCACTTTGGCCAGCC |  |  |
| VPS20 | | F | | GTCATGGCTGAATTCGACAAC | 226 | 459 |
|  |  | R | | CAGGTAGTGGCTCTTCCAAG |  |  |
| SNF7-1 | | F | | CAGGCAAGGCGCAACCAAC | 253 | 1139 |
|  |  | R | | TGACAGACATGATCTGGTGATC |  |  |
| SNF7-2 | | F | | AACATGGAGAACATGAGGCAG | 358 | 436 |
|  |  | R | | GCGCTCCTTGAGAGTGGTTC |  |  |
| SNF7-3 | | F | | CACTTGCTACACCATTCGGT | 304 | 411 |
|  |  | R | | GTTAAATGCACGCCGCAAGG |  |  |
| VPS2-1 | | F | | GAAATCGGCATCGACATCAAC | 192 | 297 |
|  |  | R | | GGATAGTCCGTCAGAGTCCAAC |  |  |
| VPS2-2 | | F | | TGCCATCGATGAAACACTAGA | 292 | 719 |
|  |  | R | | GCACTATATAAACTTTCTTGCAG |  |  |
| VPS2-3 | | F | | CTCAATAGACAATGTCTTAG | 232 | 939 |
|  |  | R | | AGAGGGTTGCAAAGACGAGT |  |  |
| VPS46 | | F | | CCCGAGACCGAGGTCAACT | 243 | 2425 |
|  |  | R | | CTTGACAACTGATGGAGAAGC |  |  |
| VPS60-1 | | F | | AGATGCTCAACAGACTATGACT | 397 | 1379* |
|  |  | R | | GGTACGAATTGACGCGTGC |  |  |
| VPS60-2 | | F | | TGAGGAAGCTGATCTTAACTTG | 172 | 574 |
|  |  | R | | CAATGAGGTAATAGTAACACTG |  |  |
| Cloning of BiFC constructs | | | | |  |  |
| HvSNF7_NcoI-F | | |  | ATACCATGGATGTTCAACAGGTTATTTG |  |  |
| HvSNF7_NotI-R | | |  | AGCGGCCGCCCAATGCCATTTCAGCTTG |  |  |
| HvVPS60_NcoI-F | | |  | ATACCATGGATGAAGAAGATCTTTG |  |  |
| HvVPS60_NotI-R | | |  | AGCGGCCGCCGGTACGAATTGACGCGTG |  |  |

*derived from aligning cDNA with genomic locations listed in Table 1

**primer F spans exon/intron border
