# Supplementary material for: Cell layer-specific distribution of transiently expressed barley ESCRT-III component HvVPS60 in developing barley endosperm
Source: Protoplasma. 2015 Mar 22;253(1):137–53. doi: 10.1007/s00709-015-0798-1 (PMC4712231; doi:10.1007/s00709-015-0798-1)
Supplement: Supplementary file 9 — Maize ESCRT-III proteins present in endosperm filtered from Dataset 1, (Walley et al. 2013) (DOCX 20 kb) [file 709_2015_798_MOESM6_ESM.docx]

Table S2

| Normalized Spectral Counts, Biological Replicates Averaged | | | | |  |  |
| --- | --- | --- | --- | --- | --- | --- |
| Endosperm  8 DAP | Endosperm 10 DAP | Endosperm 12 DAP | Endosperm Crown  27 DAP | Pericarp Aleurone 27 DAP | Gene ID | ESCRT-III member |
| 3,30587 | 2,479731 | 3,546002 | 6,41275 | 2,317262 | GRMZM2G117935 | ZmSAL1 |
| 0,697591 | 1,426658 | 2,158947 | 5,219238 | 3,369928 | GRMZM2G431900 | CHMP2.1b |
| 0,932869 | 0,915853 | 1,21789 | 2,376285 | 1,639794 | GRMZM2G069005 | CHMP2.1b* |
| 0,839219 | 0,883405 | 1,168716 | 1,800409 | 0,420923 | GRMZM2G046676 | CHMP2.2 |
| 0 | 0 | 0,394711 | 0,935992 | 0 | GRMZM2G004996 | CHMP2.3 |
| 0 | 0 | 0 | 0,427732 | 0,712341 | GRMZM2G042552 | CHMP3a |
| 2,131854 | 2,690749 | 2,513196 | 6,18949 | 4,228519 | GRMZM2G165195 | CHMP3b |
| 5,94228 | 5,892444 | 8,827761 | 26,03226 | 10,39759 | GRMZM2G107757 | CHMP4a |
| 4,965038 | 4,705481 | 3,594095 | 27,20801 | 11,09428 | GRMZM2G044805 | CHMP4b |
| 0,393876 | 0,429085 | 0 | 0,447864 | 0 | GRMZM2G103217 | CHMP4c |
| 1,120712 | 0 | 0 | 1,432028 | 0,914679 | GRMZM2G092468 | CHMP5.1 |
| 1,757466 | 2,043648 | 0,772141 | 1,92608 | 1,068168 | GRMZM2G069827 | CHMP5.2 |
| 8,333609 | 5,598499 | 1,55202 | 6,696655 | 2,966645 | GRMZM2G122983 | CHMP6 |
| 3,576759 | 2,830027 | 1,788055 | 6,099611 | 2,922475 | GRMZM2G101274 | CHMP6** |

*not included in ESCRT-III list: assigned as allele of CHMP2.1b,

**not included in ESCRT-III list: deletion of approximately AA1-30
