# Supplementary material for: Cell layer-specific distribution of transiently expressed barley ESCRT-III component HvVPS60 in developing barley endosperm
Source: Protoplasma. 2015 Mar 22;253(1):137–53. doi: 10.1007/s00709-015-0798-1 (PMC4712231; doi:10.1007/s00709-015-0798-1)
Supplement: Supplementary file 10 — Barley (Hordeum vulgare Barke) ESCRT-III genes expressed during development and germination in endosperm, filtered from Supplemental Table S2 of (Sreenivasulu et al. 2008) (DOCX 24 kb) [file 709_2015_798_MOESM7_ESM.docx]

Table S3

|  |  |  | Normalized expression values | | | | | | | | | | | | | | | |
| --- | --- | --- | --- | --- | --- | --- | --- | --- | --- | --- | --- | --- | --- | --- | --- | --- | --- | --- |
| Probe Set ID | GeneBank ID | ESCRT-III member | E4_1_daf | E4_2_daf | E8_1_daf | E8_2_daf | E16_1_daf | E16_2_daf | E25_1_daf | E25_2_daf | E0_1_hai | E0_2_hai | E24_1_hai | E24_2_hai | E48_1_hai | E48_2_hai | E72_1_hai | E72_2_hai |
| Contig23377_s_at ** | AK250448.1 | VPS2.1 | 2,88 | 2,89 | 2,52 | 2,71 | 2,79 | 2,92 | 2,50 | 3,36 | 3,45 | 3,85 | 3,37 | 3,81 | 3,65 | 3,68 | 3,70 | 3,47 |
| Contig5971_at | AK250448.1 |  | 0,42 | 0,28 | 0,18 | 0,27 | -0,07 | 0,23 | -0,89 | 0,36 | -0,10 | 0,39 | 1,03 | 0,94 | 0,70 | 0,71 | 0,39 | -0,40 |
| HT11D18u_x_at *** | AK250448.1 |  | -3,63 | -2,97 | -3,58 | -3,50 | -3,61 | -3,72 | -3,48 | -3,51 | -3,48 | -2,39 | -3,64 | -1,31 | -1,73 | -2,17 | -3,62 | -1,75 |
| Contig3956_at | AK252514.1 | SAL1 | 0,68 | 0,49 | 0,50 | 0,42 | 1,11 | -0,17 | -0,10 | 0,04 | 1,39 | 1,28 | 0,90 | 0,93 | 1,23 | 1,20 | 0,07 | 0,17 |
| Contig3958_s_at | AK252514.1 |  | 2,17 | 2,15 | 2,04 | 2,60 | 2,28 | 2,49 | 1,73 | 2,47 | 2,36 | 2,47 | 2,96 | 2,88 | 2,66 | 2,83 | 1,90 | 1,59 |
| Contig4582_at * | AK251833.1 | SNF7a | 3,12 | 3,05 | 3,10 | 3,31 | 3,70 | 3,19 | 3,69 | 4,00 | 2,78 | 2,91 | 3,28 | 3,40 | 3,30 | 3,34 | 2,98 | 2,74 |
| Contig4583_s_at | AK360610.1 | SNF7a | -0,84 | -0,55 | -0,27 | -0,03 | 0,06 | -0,42 | -0,03 | 0,28 | -2,54 | -1,94 | -1,55 | -1,41 | -0,58 | -1,16 | -1,78 | -3,25 |
| Contig7548_at * | AK367598.1 | SNF7b | 1,98 | 1,84 | 2,85 | 2,47 | 2,57 | 2,22 | 2,58 | 3,08 | 3,36 | 3,04 | 3,45 | 3,35 | 3,89 | 3,96 | 3,70 | 3,70 |
| Contig5731_at | AK364437.1 | VPS24 | 1,41 | 1,84 | 1,96 | 1,82 | 2,09 | 1,98 | 1,35 | 1,98 | 1,97 | 2,07 | 2,34 | 2,28 | 2,16 | 2,52 | 2,10 | 2,08 |
| HVSMEf0006D15r2_s_at | AK364437.1 |  | 2,55 | 2,11 | 2,60 | 2,43 | 3,18 | 2,57 | 2,12 | 3,02 | 3,65 | 3,45 | 2,70 | 3,65 | 3,65 | 3,99 | 3,30 | 3,31 |
| Contig7551_at | AK372130 | VPS60a | 2,20 | 1,89 | 1,56 | 2,07 | 2,48 | 1,94 | 2,41 | 2,70 | 1,42 | 2,12 | 2,31 | 2,34 | 2,53 | 2,68 | 2,35 | 2,32 |
| Contig16807_at | AK367793 | VPS60b | 0,82 | 0,79 | 0,30 | 0,54 | -0,38 | -0,32 | -0,54 | -0,47 | -0,48 | -0,92 | -1,23 | -1,53 | -0,57 | -1,06 | 0,15 | -0,55 |
| Contig14049_at | AK367793 |  | 1,19 | 1,35 | 0,63 | 1,57 | -3,61 | -3,72 | -3,48 | -3,54 | -3,48 | -3,38 | -3,64 | -3,36 | -2,76 | -3,31 | -3,26 | -3,61 |
|  |  | Other ESCRT members |  |  |  |  |  |  |  |  |  |  |  |  |  |  |  |  |
| Contig15210_at | AK365676.1 | VPS28  (ESCRT I) | 2,33 | 2,25 | 2,08 | 2,26 | 3,18 | 3,27 | 3,53 | 3,41 | 2,79 | 2,99 | 2,77 | 3,02 | 2,63 | 2,87 | 2,36 | 2,32 |
| Contig19916_at | AK354992.1 | EAP30/Vps36 (ESCRTII) | -0,98 | -0,49 | -0,44 | -0,44 | -0,06 | 0,33 | 0,32 | 0,40 | -3,15 | -1,61 | -2,51 | -3,36 | -1,15 | -0,65 | -2,82 | -2,52 |
| HVSMEa0014K05r2_at | AK354992.1 |  | -1,49 | -1,68 | -1,19 | -1,89 | -1,01 | -0,83 | -0,07 | -0,66 | -1,09 | -1,30 | -1,46 | -1,22 | -0,97 | -0,15 | -0,65 | -0,61 |

*probe sets interrogating splice isoforms to respective SNF7 cDNAs in ESCRT-III inventory affecting 3´UTR

**probe set with microstaellite sequence not present at VPS2.1 locus, a BLAST search of barley genomic sequences does not find sequence

***probe set with polymorphisms to VPS2.1 Haruna nijo; however also not identical to Bowman, Barke, Morex alleles
